# Integrative Network Pharmacology and Transcriptomics Reveal the Mechanism of XuanShi Choudong Recipe in the Treatment of Tic Disorder

**DOI:** 10.1002/brb3.71687

**Published:** 2026-08-17

**Authors:** Hu Jue, Ma Jingying, Chen Yaqin, Pan Yulu, Chen Yanhong, Xu Jiaye, Chen Danfei, Chen Jian, Xuan Xiao‐bo

**Affiliations:** ^1^ Department of Pediatrics, The First Affiliated Hospital of Zhejiang Chinese Medical University (Zhejiang Provincial Hospital of Chinese Medicine) Zhejiang Chinese Medical University Hangzhou China; ^2^ Department of Clinical Psychology Hangzhou TCM Hospital Affiliated to Zhejiang Chinese Medical University Hangzhou China; ^3^ Laboratory Animal Center Zhejiang University Hangzhou China

**Keywords:** apoptosis, neurotransmitters, oxidative stress injury, PI3K/AKT/Bcl‐2 signaling pathway, TD, XuanShi Choudong Recipe

## Abstract

**Ethnopharmacological relevance:**

The Xuanshi Choudong Recipe (CDR), a classic traditional Chinese medicine (TCM) formula, has long been clinically prescribed to relieve tic disorder (TD). However, its underlying in‐depth mechanism remains largely unknown.

**Aim of the study:**

To elucidate the mechanism by which CDR exerts therapeutic effects in the treatment of TD.

**Materials and methods:**

Four‐week‐old SD rats were injected intraperitoneally with apomorphine at 2 mg/kg per day to establish the model. After successful modeling, the rats were randomly divided into the model, CDR low‐dose (CDRL, 6.84 g/kg), CDR medium‐dose (CDRM, 13.67 g/kg), CDR high‐dose (CDRH, 27.34 g/kg), and haloperidol (1 mg/kg) groups. Age‐matched SD rats served as the control group. Therapeutic efficacy was evaluated using the open field test, hematoxylin and eosin (H&E) staining, and biochemical analysis. The potential mechanisms of CDR were explored using network pharmacology and transcriptomic analyses and validated by western blotting, immunofluorescence, biochemical analysis, and terminal deoxynucleotidyl transferase dUTP nick‐end labeling (TUNEL) staining.

**Results:**

In this study, a total of 126 compounds were identified in CDR using liquid chromatography‐mass spectrometry, with phenylpropanoids, polyketides, benzenoids, and lipids being the main components. Network pharmacology prediction revealed 265 overlapping targets between CDR and TD, with core targets including AKT1, BCL2, and IL6, and the phosphoinositide 3‐kinase/protein kinase B (PI3K‐AKT) signaling pathway being significantly enriched. Transcriptomic analysis showed that CDR reversed the expression of 111 genes in the striatum of TD rats, and these genes were significantly enriched in pathways related to apoptosis and the PI3K‐Akt signaling pathway. Behavioral assessment demonstrated that CDR dose‐dependently reduced apo‐induced stereotypic behavior scores and spontaneous locomotor activity in TD rats. H&E staining indicated that CDR attenuated striatal neuronal damage. High dose CDR treatment significantly decreased the elevated levels of dopamine, 5‐HT, norepinephrine, glutamate, and gamma‐aminobutyric acid in the striatum (*p* < 0.01 or *p* < 0.001). Furthermore, High‐dose CDR alleviated oxidative stress‐induced injury by reducing reactive oxygen species and oxidized glutathione (GSSG) levels and increasing superoxide dismutase (SOD) activity, reduced glutathione (GSH) levels, and the GSH/GSSG ratio (*p* < 0.01 or *p* < 0.001). TUNEL staining and immunofluorescence results showed that CDR inhibited neuronal apoptosis in the striatum. Western blotting confirmed that CDR upregulated the phosphorylation levels of PI3K and AKT, increased the Bcl‐2/Bax ratio, and downregulated caspase‐3 expression, with high‐dose CDR exerting the most significant effect.

## Graphical abstract

Using an integrated strategy of network pharmacology and transcriptomics, this study elucidates the therapeutic mechanism of XuanShi Choudong Recipe (CDR) against tic disorder (TD). CDR dose‐dependently alleviates apomorphine‐induced tic‐like stereotypy and hyperlocomotion in rats, restores striatal neurotransmitter homeostasis, and attenuates oxidative stress injury and neuronal apoptosis. Mechanistically, the neuroprotective effect of CDR is closely associated with activation of the PI3K/AKT/Bcl‐2 signaling pathway, providing preliminary experimental evidence for its clinical application in TD.

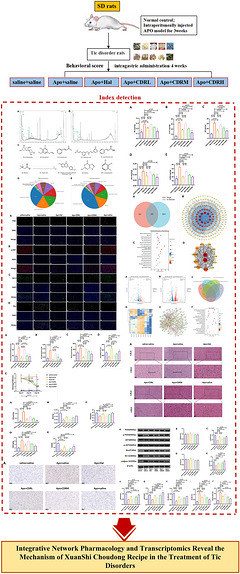

**Abbreviations**
5‐HT: serotonin
AKT: protein kinase B
APO: apomorphine
BBB: blood–brain barrier
Bcl‐2: B‐cell lymphoma 2
BPC: base‐peak chromatogram
CDR: XuanShi Choudong Recipe
CDRH: high‐dose CDR
CDRL: low‐dose CDR
CDRM: medium‐dose CDR
DA: dopamine
DAPI: 4′,6‐diamidino‐2‐phenylindole
DEG: differentially expressed gene
DRD1: dopamine receptor D1
DRD2: dopamine receptor D2
ELISA: enzyme‐linked immunosorbent assay
GABA: γ‐aminobutyric acid
Glu: glutamate
GO: Gene Ontology
GSH: reduced glutathione
GSSG: oxidized glutathione
H&E: hematoxylin and eosin
HAL: haloperidol
HMDB: Human Metabolome Database
HRP: horseradish peroxidase
IHC: immunohistochemistry
KEGG: Kyoto Encyclopedia of Genes and Genomes
LC–MS: liquid chromatography–mass spectrometry
NE: norepinephrine
OMIM: Online Mendelian Inheritance in Man
PBS: phosphate‐buffered saline
PI3K: phosphoinositide 3‐kinase
PPI: protein–protein interaction
ROS: reactive oxygen species
SD: standard deviation
SOD: superoxide dismutase
SPF: specific pathogen‐free
TCM: traditional Chinese medicine
TD: tic disorder
TUNEL: terminal deoxynucleotidyl transferase dUTP nick‐end labeling
UPLC: ultra‐performance liquid chromatography
WB: western blotting

## 1 Background

Tic disorders (TD) are common childhood‐onset neurodevelopmental disorders characterized by sudden, rapid, recurrent, nonrhythmic motor movements and/or vocalizations (Woods et al. 2023). Their etiology remains incompletely understood and likely involves interacting genetic, neurobiological, psychological, and environmental factors. Among the proposed mechanisms, dysregulation of neurotransmitter systems—particularly excessive dopaminergic activity or altered dopamine receptor sensitivity within the basal ganglia and striatum—has been widely implicated (Wang et al. 2012). Traditional Chinese medicine (TCM) has increasingly been investigated as a treatment option for TD and may provide symptomatic benefit with a relatively favorable tolerability profile (Kong et al. 2023).

Xuan's Pediatrics, a traditional medical lineage established in Hangzhou during the late Qing Dynasty, has long been associated with the treatment of pediatric tic disorder. Professor Xuan Guiqi, its third‐generation successor and a nationally recognized practitioner of TCM, developed XuanShi Choudong Recipe (CDR) as an empirical formula for TD. CDR comprises 10 medicinal materials: *Os Draconis*, *Paeonia lactiflora* Pall., *Poria cocos (Schw.)* Wolf., *Gastrodia elata* Blume, *Haliotis diversicolor* Reeve, *Cryptotympana pustulata* Fabricius, *Buthus martensii* Karsch, *Curcuma wenyujin* Y. H. Chen et C. Ling, and *Acorus tatarinowii* Schott, *Arisaema erubescens* Schott. The herb's name has been checked with “World Flora Online,” MPNS, or the Chinese pharmacopeia. The formula was derived from Dingxian Wan, a classical prescription traditionally used for convulsive disorders and first recorded in Yixue Xinwu in 1732 (Guopeng 1959). Experimental studies have reported sedative, anticonvulsant, or neurotransmitter‐modulating activities for several constituents of CDR. *Os Draconis* has been associated with central inhibitory, sedative, and antispasmodic effects (Zhang et al. 2011); *Buthus martensii* Karsch and *Cryptotympana pustulata* Fabricius have shown anticonvulsant or neuroactive properties (He et al. 2023; Xiao et al. 2022); and *Gastrodia elata* Blume may modulate cerebral dopamine levels (Zhang and Li 2015). *Acorus tatarinowii* Schott and *Curcuma wenyujin* have also been reported to possess neuropharmacological activities (Li et al. 2021; Feng et al. 2021; Wang et al. 2023). Guided by the TCM principle of calming the liver to extinguish wind and resolving phlegm to open the orifices, CDR has shown potential clinical benefit and acceptable tolerability in preliminary studies of pediatric TD. Previous work suggested that its effects may be related to changes in circulating epinephrine and dopamine and to regulation of γ‐aminobutyric acid (GABA) and striatal dopamine receptor expression (Xu et al. 2025). Clinical observations have also reported improvements in tic symptoms and related impairment compared with tiapride, although further rigorously designed trials are required (Li 2019; Yang et al. 2018). Nevertheless, the transcriptome‐level effects of CDR and the signaling pathways potentially associated with its neuroprotective activity have not been systematically characterized. In the present study, a rat model of tic‐like stereotypy was established by intraperitoneal administration of apomorphine (APO), a dopamine D1/D2 receptor agonist that induces hyperlocomotion and repetitive stereotyped behaviors, including sniffing, licking, and biting (Müller‐Vahl et al. 2005). We integrated network pharmacology, transcriptomic sequencing, and multidimensional in vivo validation to investigate the potential mechanisms by which CDR ameliorates tic‐like phenotypes. The findings are intended to provide preliminary experimental support for subsequent mechanistic and translational studies.

## 2 Materials and Methods

### 2.1 Drugs

CDR was supplied by Zhejiang Provincial Hospital of Chinese Medicine. Apomorphine (APO; batch no. G2205058) and haloperidol (HAL; batch no. G1411016) were purchased from Shanghai Aladdin Biochemical Technology Co., Ltd. (Shanghai, China).

### 2.2 Component Analysis

As shown in Table 1, CDR contains 10 medicinal materials of botanical, fungal, animal, and mineral origin: *Os Draconis* (10 g; Ningxia; batch no. 211218), *Paeonia lactiflora* Pall. root (6 g; Anhui; batch no. 220610), *Poria cocos (Schw.)* Wolf sclerotium (10 g; Anhui; batch no. 220528), *Gastrodia elata* Blume rhizome (5 g; Anhui; batch no. 220528), *Haliotis diversicolor* Reeve shell (10 g; Guangdong; batch no. 220602), *Cryptotympana pustulata* Fabricius slough (6 g; Zhejiang; batch no. 20221101), *Buthus martensii* Karsch dried body (3 g; Shandong; batch no. 220515), *Curcuma wenyujin* Y.H. Chen & C. Ling tuberous root (5 g; Zhejiang; batch no. 210902), *Acorus tatarinowii* Schott rhizome (5 g; Zhejiang; batch no. 220417), and *Arisaema erubescens* Schott processed with bile (5 g; Zhejiang; batch no. 211111). The Pharmacy Department of Zhejiang Provincial Hospital of Chinese Medicine prepared the decoction. The medicinal materials were soaked in 1000 mL of distilled water for 20 min and decocted for 60 min. Following filtration, the remaining material underwent a second 60‐min extraction with the same volume of distilled water. Both extracts were pooled and concentrated in a water bath to 6.5 g crude drug/mL. The final preparation was refrigerated at 4°C until administration.

**TABLE 1:** Composition of XuanShi Choudong Recipe.

Component	Part used	Amount (g)
*Os Draconis*	Teeth/fossil material	10
*Paeonia lactiflora* Pall.	Root	6
*Poria cocos (Schw.)* Wolf.	Sclerotium	10
*Gastrodia elata* Blume	Rhizome	5
*Haliotis diversicolor* Reeve	Shell	10
*Cryptotympana pustulata* Fabricius	Slough (exuviae)	6
*Buthus martensii* Karsch	Dried body	3
*Curcuma wenyujin* Y. H. Chen et C. Ling	Tuberous root	5
*Acorus tatarinowii* Schott	Rhizome	5
*Arisaema erubescens* Schott	Tuber processed with bile	5

#### 2.2.1 Liquid Chromatography–Mass Spectrometry Analysis

Chemical constituents were profiled with a Synapt G2‐Si high‐resolution quadrupole time‐of‐flight tandem mass spectrometer coupled to liquid chromatography. Separation was performed on an ACQUITY UPLC HSS T3 column. The aqueous mobile phase (A) and acetonitrile mobile phase (B) each contained 0.1% formic acid, and the flow rate was 0.35 mL/min. Samples (2 µL) were injected while the column was held at 45°C. The gradient consisted of 5% B at 0–1 min, 5%‐30% B at 1–4 min, 30%‐50% B at 4–8 min, 50%‐80% B at 8–10 min, 80%‐100% B at 10–14 min, 100% B at 14–15 min, and 100%‐5% B at 15‐15.1 min, followed by re‐equilibration at 5% B to 16 min. mass spectra were recorded in positive‐ and negative‐ion modes across m/z 100–1200. Source conditions comprised a sheath‐gas flow of 35; an auxiliary‐gas flow of 8, spray voltages of +3.8 kV and ‐3.2 kV for positive and negative ionization, respectively; and a capillary temperature of 320°C. Both sensitivity and resolution acquisition modes were used.

#### 2.2.2 LC–MS Data Preprocessing and Compound Annotation

Raw LC–MS data were preprocessed using Progenesis QI software (version 2.3; Nonlinear Dynamics, Newcastle upon Tyne, UK). The workflow included baseline correction, peak detection and deconvolution, retention‐time alignment, peak‐area integration, and signal normalization. A feature matrix containing the mass‐to‐charge ratio (m/z), retention time, and peak intensity was generated for subsequent statistical analysis and feature screening. Compounds were annotated using Compound Discoverer software together with the mzVault VS1.0 local MS/MS spectral library. Annotation confidence was evaluated using the mzVault composite matching score (Total Score), which integrates precursor‐ion matching (weight, 0.25), retention‐time similarity (weight, 0.25), and MS/MS spectral concordance (weight, 0.50). Spectral concordance was assessed using the forward dot‐product score, reverse dot‐product score, and fragment‐ion matching rate. Only compounds with a Total Score >73 that also met the predefined criteria for retention‐time agreement, accurate‐mass matching, and MS/MS fragment matching were retained. Because a composite score ≥70 is generally considered to indicate relatively high‐confidence annotation within the mzVault matching framework, the more stringent threshold used here was intended to reduce false‐positive assignments. Annotated compounds were cross‐referenced against the Human Metabolome Database (HMDB) (Wishart et al. 2018), METLIN (Xue et al. 2020), and LIPID MAPS (Sud et al. 2012) to obtain complementary information on chemical structures and classifications.

### 2.3 Network Pharmacology Analysis

Compounds identified in the aqueous CDR decoction were evaluated in silico for gastrointestinal absorption, blood–brain barrier (BBB) permeability, and oral bioavailability using SwissADME (https://www.swissadme.ch/) (Daina et al. 2017). The BOILED‐Egg model was used to predict passive gastrointestinal absorption and BBB penetration. Compounds were retained for network pharmacology analysis when they met all of the following criteria: (1) high predicted gastrointestinal absorption; (2) predicted BBB permeability; and (3) a bioavailability score ≥0.55, indicating moderate‐to‐high oral bioavailability potential (Daina and Zoete 2016). The retained compounds were subsequently mapped to putative protein targets. TD‐associated genes were retrieved from GeneCards (https://www.genecards.org) and Online Mendelian Inheritance in Man (OMIM; https://www.omim.org) using the search terms “tic disorder” and “Tourette syndrome” (Amberger et al. 2015; Stelzer et al. 2016) (Amberger et al. 2015; Stelzer et al. 2016). Duplicate entries were removed, and gene symbols were standardized according to the HUGO Gene Nomenclature Committee nomenclature.

### 2.4 Experimental Animals and Treatment

Thirty‐six male Sprague–Dawley rats (3 weeks old; 70–80 g) were obtained from Shanghai Bikai Keyi Biotechnology Co., Ltd. (production license no. SCXK [Hu] 2023‐0009; animal use license no. SYXK [Zhe] 2021‐0012; ethics approval no. IACUC‐20220822‐07). Animals were housed in a specific pathogen‐free facility at the Animal Center of Zhejiang Chinese Medical University under controlled conditions (20–24°C, 40%–70% relative humidity, a 12‐h light/12‐h dark cycle, and 10–20 air changes per hour), with ad libitum access to food and water. After 1 week of acclimatization, the rats were randomly assigned to a control group (*n* = 6) or a modeling cohort (*n* = 30).

Rats in the modeling cohort received intraperitoneal APO at 2 mg/kg once daily for 21 consecutive days to induce tic‐like stereotypy, whereas control rats received an equal volume of normal saline (Ke and Chen, 2024). Injections were administered between 09:00 AM and 10:00 AM. After successful model establishment, the modeled rats were randomly assigned using SPSS software to the model, CDR low‐dose (CDRL), CDR medium‐dose (CDRM), CDR high‐dose (CDRH), or APO + HAL group (*n* = 6 per group). CDRL, CDRM, and CDRH rats received CDR by gavage at 6.84, 13.67, and 27.34 g/kg/day, respectively. The APO + HAL group received haloperidol at 1 mg/kg/day (Zhou et al. 2009), and the model and control groups received normal saline at 10 mL/kg/day. Treatments were administered once daily between 09:00 and 10:00 for 28 days. The CDR doses were calculated by body‐surface‐area conversion from a clinical daily dose of 65 g for a 20‐kg child (Reagan‐Shaw et al. 2008). Using the Meeh–Rubner formula, the body surface area of a 170‐g rat was estimated to be approximately 0.0279 m^2^. Accordingly, 13.67 g/kg/day was defined as the clinical‐equivalent dose, 27.34 g/kg/day as twice the clinical‐equivalent dose, and 6.84 g/kg/day as half the clinical‐equivalent dose. Model establishment was evaluated using stereotypic behavior scores beginning 5 min after APO administration. Rats were allowed to acclimate in a quiet, dimly lit environment for 30 min. Two investigators who were blinded to group allocation independently observed each rat for 1–2 min and assigned a score; the mean of the two scores was used for analysis. Model establishment was considered successful when the mean score was ≥2. The scoring criteria were as follows: 0, no behavioral difference from saline‐treated rats; 1, intermittent sniffing, often accompanied by increased activity; 2, continuous sniffing and mild head movements with periodic hyperactivity; 3, continuous sniffing and frequent head movements accompanied by intermittent biting, gnawing, or licking and brief periods of hyperactivity; and 4, continuous biting, gnawing, and licking without intervals of hyperactivity, occasionally accompanied by rapid whole‐body movements (Lv et al. 2009). At the end of the study, the rats were euthanized, and striatal tissues were collected for subsequent analyses.

### 2.5 Open‐Field Test

Open‐field behavior was assessed in a black 50 cm × 50 cm chamber with a camera mounted directly above the arena. On the day before testing, rats were allowed to explore the chamber for 30 min to reduce novelty‐related activity. Before each trial, the arena was cleaned with 75% ethanol and allowed to dry. Each rat was then permitted to move freely for 10 min. Total distance traveled (cm), mean speed (cm/s), movement duration (s), and immobility duration (s) were quantified using the SMART 3.0 video‐tracking system (Harvard Apparatus/Panlab, USA).

### 2.6 Transcriptomic Analysis

Total RNA was extracted from striatal tissue using TRIzol reagent. RNA quantity and integrity were assessed using a NanoDrop 2000 spectrophotometer and an Agilent 2100 Bioanalyzer. Three biological replicates per group were used for library preparation with the VAHTS Universal V6 RNA‐seq Library Prep Kit, followed by paired‐end high‐throughput sequencing on a BGI/Illumina platform. Raw reads were filtered using fastp, and clean reads were aligned to the Rattus norvegicus reference genome (Rnor_6.0) using STAR. Gene‐level read counts were generated using HTSeq, and fragments per kilobase of transcript per million mapped reads (FPKM) values were calculated for expression visualization. Differential expression analysis was performed using DESeq2 on the count data. Genes with |log_2_ fold change| > 0.585 and an adjusted *p*‐value < 0.05 were considered differentially expressed. Gene Ontology (GO) and Kyoto Encyclopedia of Genes and Genomes (KEGG) enrichment analyses were performed using clusterProfiler. A total of 96.61 Gb of clean data was generated; all samples had Q30 values ≥96.01% and genome‐mapping rates of 97.14%–97.73%, indicating acceptable sequencing quality. Additional experimental and bioinformatic details, including RNA extraction and quality control, library construction, raw data processing, genome alignment, differential expression analysis, and functional enrichment procedures, are provided in Supplementary Methods 1.

### 2.7 Hematoxylin and Eosin Staining

After perfusion, brain tissues were fixed in 4% paraformaldehyde, embedded in paraffin, and sectioned. Striatal sections were stained with hematoxylin and eosin (H&E) and scanned using a Pannoramic slide‐scanning system (3DHISTECH, Hungary). Morphologically intact neurons were defined by a rounded, well‐preserved cell body and a clearly visible nucleus. Images were analyzed using ImageJ, and the number of morphologically intact neurons in the striatum was quantified.

### 2.8 Immunohistochemical Staining

Paraffin sections were deparaffinized and rehydrated before antigen retrieval. Endogenous peroxidase was quenched with 3% H2O2, and goat serum was used to minimize nonspecific binding. Sections were exposed overnight at 4°C to antibodies against dopamine receptor D1 (DRD1; 1:4000; Abcam, ab279713) or dopamine receptor D2 (DRD2; 1:200; Proteintech, 22022‐1‐AP). After returning to room temperature, horseradish peroxidase‐conjugated goat anti‐rabbit IgG (1:2,000; Abcam, ab205718) was applied for 1 h. Signals were developed with 3,3′‐diaminobenzidine. Slides were counterstained with hematoxylin, dehydrated, cleared, mounted in neutral balsam, and scanned on a Pannoramic MIDI system (3DHISTECH, Hungary).

### 2.9 Measurement of Oxidative Stress Markers and Neurotransmitters

Three animals from each group were randomly selected for biochemical measurements. Striatal tissue was combined with extraction buffer and homogenized on ice. Following centrifugation at 4,000 × g for 10 min, the supernatant was retained. Protein content was measured with a bicinchoninic acid kit (Shanghai Weiao Biotechnology Co., Ltd.; WH0124). reactive oxygen species (ROS) were quantified with the kit ET0008S from the oxidation‐dependent absorbance change of a chromogenic substrate. Superoxide dismutase (SOD) activity was determined with the kit ET0012S by measuring inhibition of nitroblue tetrazolium reduction. Kits ET0003S and ET0004S were used for reduced glutathione (GSH) and oxidized glutathione (GSSG), and the GSH/GSSG ratio was subsequently calculated. Enzyme‐linked immunosorbent assays measured glutamate (ET0002S), norepinephrine (ER20535S), GABA (ER20934S), dopamine (ER20529S), and serotonin (ER20533S) (Shanghai Weiao Biotechnology Co., Ltd.). Procedures followed the suppliers’ protocols. Absorbance readings were obtained with a microplate reader, and concentrations were interpolated from standard curves.

### 2.10 Detection of Apoptosis by Terminal Deoxynucleotidyl Transferase dUTP Nick‐End Labeling

Apoptotic DNA fragmentation was evaluated with a TUNEL kit using 3,3′‐diaminobenzidine detection (Wuhan Servicebio Technology Co., Ltd.; GDP1047). Paraffin sections were cleared in xylene, passed through graded ethanol, and incubated with working proteinase K for 30 min at 37°C. After phosphate‐buffered saline washes, a reaction mixture containing terminal deoxynucleotidyl transferase and digoxigenin‐labeled dUTP was applied for 2 h at 37°C in a humidified chamber. Sections were washed again and treated for 30 min at 37°C with streptavidin‐horseradish peroxidase. Color development with 3,3′‐diaminobenzidine was monitored microscopically and stopped with distilled water. Hematoxylin was used for nuclear counterstaining, after which slides were dehydrated, cleared, and mounted. Positive nuclei appeared brown‐yellow and negative nuclei blue. The apoptotic index was the percentage of TUNEL‐positive nuclei among the total nuclei evaluated.

### 2.11 Immunofluorescence Staining

Deparaffinized and rehydrated sections were permeabilized and blocked, then incubated separately with antibodies to PI3K (1:200; Abcam, ab191606), phosphorylated AKT (p‐AKT; 1:200; Abcam, ab8805), Bcl‐2 (1:200; Abcam, ab194583), or Bax (1:200; Abcam, ab263897). Alexa Fluor 594‐conjugated goat anti‐rabbit IgG (1:800; Abcam, ab150080) served as the secondary antibody. Nuclei were labeled with 4′,6‐diamidino‐2‐phenylindole (DAPI; Beijing Solarbio Science & Technology Co., Ltd.). Slides were mounted in antifade medium and scanned with the Pannoramic MIDI platform (3DHISTECH, Hungary).

### 2.12 Western Blotting

Approximately 30 mg of striatal tissue was homogenized in PBS and centrifuged for 15 min at 12,000 × g and 4°C. Protein in the supernatant was quantified by bicinchoninic acid assay (Elabscience, China). Equal protein loads were resolved by sodium dodecyl sulfate‐polyacrylamide gel electrophoresis and transferred onto polyvinylidene difluoride membranes. Primary antibodies were directed against PI3K (1:1000; Abcam, ab191606), p‐PI3K (1:1000; Abcam, ab182651), AKT (1:1,000; Abcam, ab300473), p‐AKT (1:1000; Abcam, ab81283), caspase‐3 (1:2000; Abcam, ab184787), Bcl‐2 (1:1000; Abcam, ab194583), Bax (1:1000; Abcam, ab263897), and beta‐actin (1:2000). The next day, membranes were exposed for 1 h at room temperature to horseradish peroxidase‐linked anti‐rabbit secondary antibody (1:5000; Proteintech, SA00001‐2). Bands were detected by enhanced chemiluminescence (Beyotime, China) and densitometrically analyzed in ImageJ.

### 2.13 Statistical Analysis

Statistical analyses were performed using SPSS version 26.0 (IBM/SPSS Inc., Chicago, IL, USA), and graphs were generated using GraphPad Prism version 9.0 (GraphPad Software Inc., La Jolla, CA, USA). Normality and homogeneity of variance were assessed using the Shapiro–Wilk and Levene tests, respectively. Normally distributed data with homogeneous variances are presented as the mean ± standard deviation (SD). Comparisons among multiple groups were performed using one‐way analysis of variance, followed by the least significant difference test for pairwise comparisons. Both technical and biological replicates were set for each experiment, with the corresponding numbers detailed in the respective figure legends. The number of biological replicates is specified in the figure legends (*n* = 6 for behavioral analyses and *n* = 3 for histological, biochemical, and molecular analyses). All tests were two‐sided, and *p* < 0.05 was considered statistically significant. Post hoc power analyses of the primary behavioral outcomes were performed using G*Power version 3.1 (Heinrich Heine University Düsseldorf, Germany) based on omega‐squared effect sizes calculated from the observed data. For outcomes with six biological replicates per group, the achieved power (1–β) for the overall one‐way analysis of variance exceeded 0.80. The molecular and biochemical assays with three biological replicates per group were considered exploratory mechanistic analyses.

## 3 Results

Using an integrated strategy combining network pharmacology, transcriptomic analysis, and in vivo validation, we found that CDR attenuated tic‐like behaviors in rats. The observed effects were accompanied by reduced oxidative stress and neuronal apoptosis, changes in striatal neurotransmitter levels, and increased phosphorylation of proteins in the PI3K/AKT/Bcl‐2 signaling axis. The detailed results are presented below.

### 3.1 Chemical Characterization of CDR by LC–MS

**FIGURE 1:**
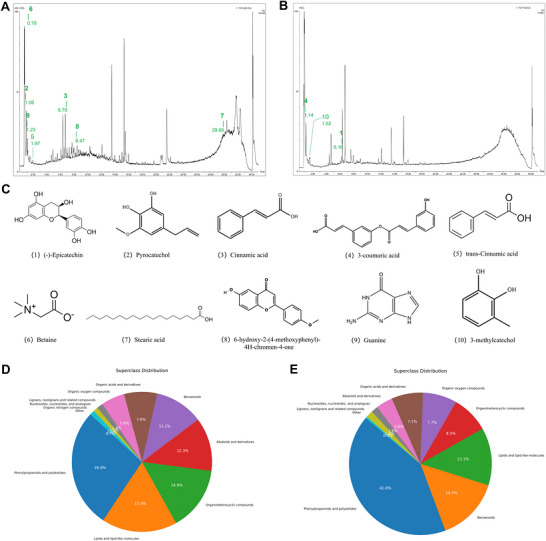
Chemical characterization of XuanShi Choudong Recipe (CDR) by liquid chromatography–mass spectrometry. (A) Chromatographic profile acquired in positive‐ion mode. (B) Chromatographic profile acquired in negative‐ion mode. (C) Chemical structures of 10 representative compounds detected in CDR. (D) Source attribution and structural classification of compounds detected in positive‐ion mode. (E) Source attribution and structural classification of compounds detected in negative‐ion mode. No technical replicates were set for this qualitative screening experiment. This assay was performed as a qualitative constituent profiling study with a single injection of the pooled CDR decoction sample using high‐resolution Q‐TOF mass spectrometry to provide a candidate compound library for subsequent network pharmacology analysis. No technical replicates were set for this qualitative screening experiment.

Liquid chromatography–tandem mass spectrometry was used to characterize the chemical constituents of CDR, as described in the Materials and Methods section. A total of 126 compounds were putatively identified; detailed annotation data are provided in Supplementary Table S1. Representative chromatographic profiles of CDR acquired in positive‐ and negative‐ion modes are shown in Figures 1A **and** 1B, respectively, and the structures of 10 representative compounds are shown in Figure 1C and Table 2. Source attribution and structural classification indicated that phenylpropanoids and polyketides, benzenoids, lipids and lipid‐like molecules, and organoheterocyclic compounds were among the predominant chemical classes identified in CDR (Figures 1D **and** 1E).

**TABLE 2:** Primary bioactive compound characterization in CDR.

No	Compound name	CAS no.	Ret.Time (min)		Ion mode	Measured value	Theoretical value	Adducts
1	(‐)‐Epicatechin	490‐46‐0	6.1648		NEG	289.0720	289.0718	[M‐H]‐
2	Pyrocatechol	4055‐72‐5	1.0647		POS	109.0296	109.0295	[M+H]+
3	Cinnamic acid	140‐10‐3	6.7763		POS	147.0450	147.0452	[M+H]+
4	3‐coumaric acid	14755‐02‐3	1.1397		NEG	163.0405	163.0401	[M‐H]‐
5	trans‐Cinnamic acid	140‐10‐3	1.9683		POS	147.0450	147.0452	[M+Na]+
6	Betaine	107‐43‐7	0.7804		POS	118.0868	118.0863	[M+H]+
7	Stearic acid	57‐11‐4	29.6585		POS	283.2643	283.2643	[M+Na]+
8	6‐hydroxy‐2‐(4‐methoxyphenyl)‐4H‐chromen‐4‐one	35794‐88‐8	8.4672		POS	269.0829	269.0820	[M+H]+
9	Guanine	73‐40‐5	1.2282		POS	152.0569	152.0567	[M+H]+
10	3‐methylcatechol	488‐17‐5	1.5205	NEG		123.0445	123.0440	[M‐H]‐

### 3.2 Behavioral Effects of CDR

**FIGURE 2:**
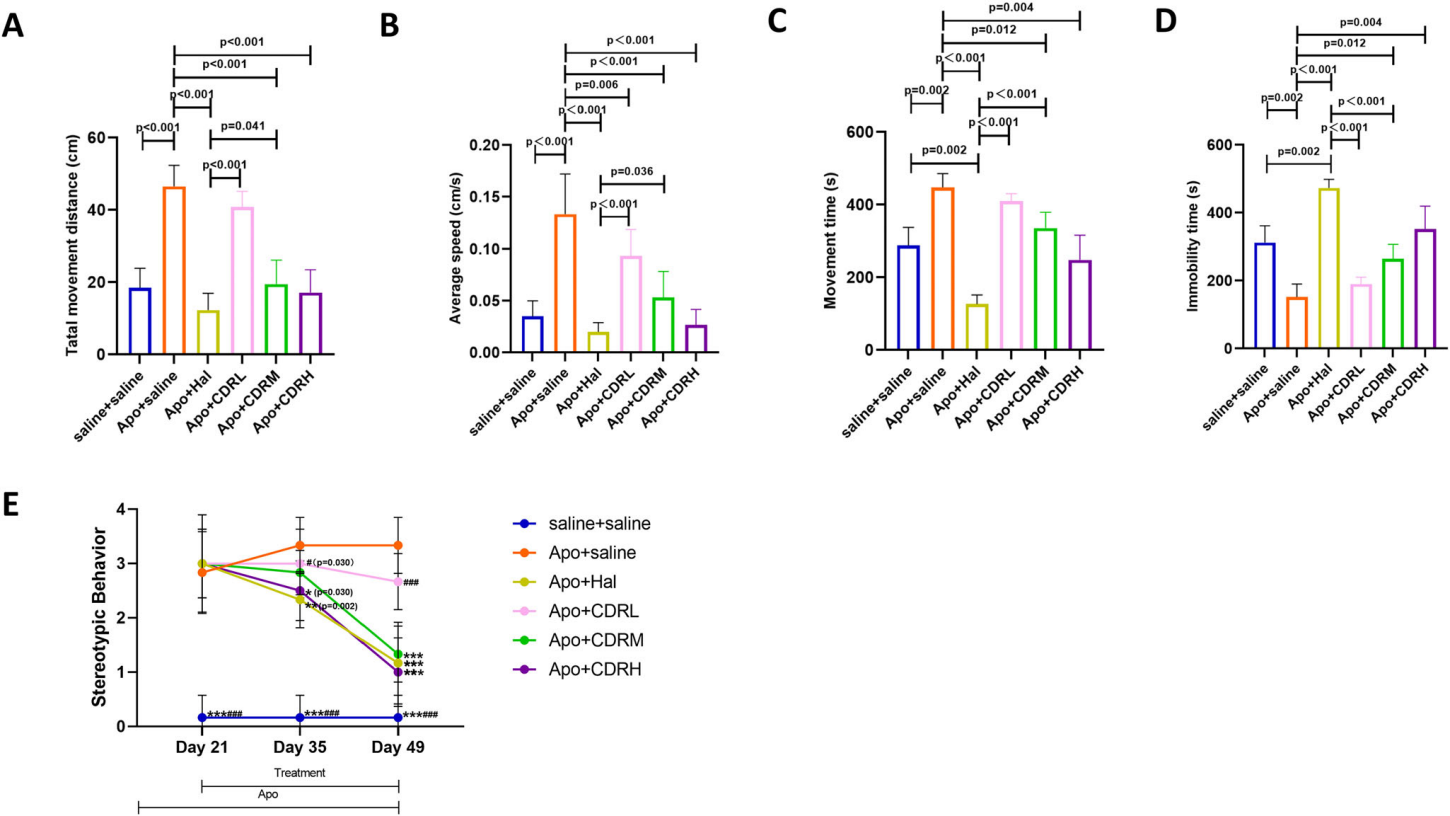
Open‐field outcomes and stereotypic behavior scores after CDR treatment. (A) Total distance traveled. (B) Mean movement speed. (C) Movement duration. (D) Immobility duration. (E) Stereotypic behavior scores after model establishment and after 2 and 4 weeks of treatment. Data are presented as the mean ± SD; *n* = 6 biological replicates per group. ^***^ *p* < 0.05, ^**^ *p* < 0.01, and ^*^ *p* < 0.001 versus the APO + saline group; ^#^ *p* < 0.05, ^##^ *p* < 0.01, and ^###^ *p* < 0.001 versus the APO + HAL group.

Open‐field outcomes after treatment are shown in Figures 2A–D. Compared with the APO + saline group, the APO + HAL, APO + CDRM, and APO + CDRH groups showed significantly shorter total distances traveled, shorter movement durations, and lower mean speeds (*p* < 0.05, *p* < 0.01, or *p* < 0.001), together with longer immobility durations (*p* < 0.05 or *p* < 0.01). None of these variables differed significantly between the APO + CDRL and APO + saline groups. The APO + CDRH and APO + HAL groups did not differ significantly in any open‐field variable, and mean speed did not differ between the APO + CDRM and APO + HAL groups (*p* > 0.05). Stereotypic behavior scores after model establishment and after 2 and 4 weeks of treatment are shown in Figure 2E. After APO administration, all modeled groups had mean scores >2, and their scores were significantly higher than those of the saline + saline group (*p* < 0.001), supporting successful model establishment. After 2 weeks of treatment, scores tended to decrease and were significantly lower in the APO + HAL and APO + CDRH groups than in the APO + saline group (p = 0.002 and p = 0.040, respectively). After 4 weeks, stereotypic behavior scores were significantly lower in the APO + HAL, APO + CDRM, and APO + CDRH groups than in the APO + saline group (all *p* < 0.001). Scores in the medium‐ and high‐dose CDR groups did not differ significantly from those in the APO + HAL group (*p* > 0.05).

### 3.3 CDR Attenuates Pathological Changes in the Striatum of TD Model Rats

**FIGURE 3:**
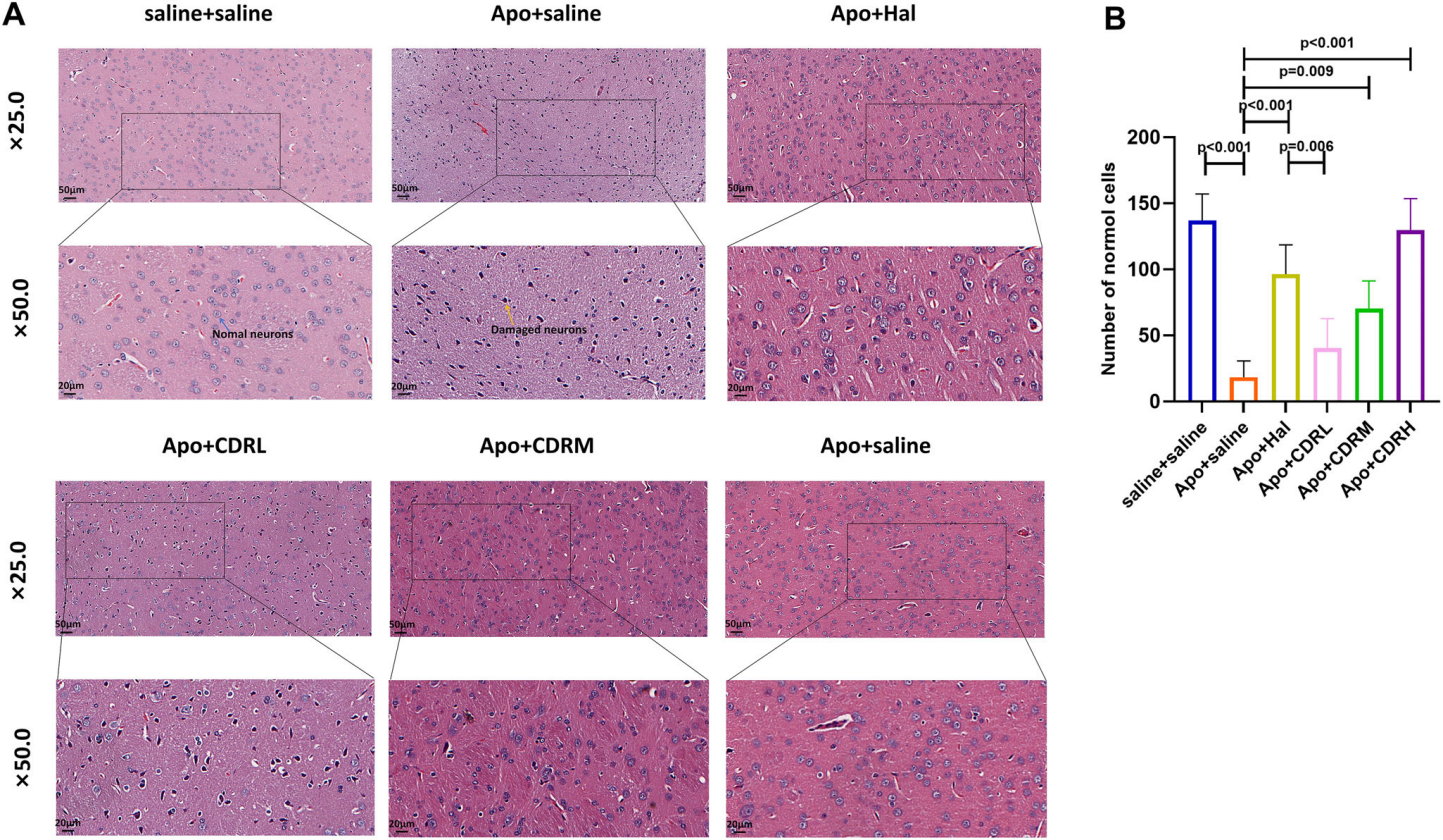
Histopathological changes in the striatum assessed by hematoxylin and eosin staining. (A) Representative striatal sections (original magnification, ×25; scale bar = 50 µm; and ×50; scale bar = 20 µm). Blue arrows indicate morphologically intact neurons with rounded cell bodies and clear nuclei; yellow arrows indicate injured cells with intense staining, nuclear pyknosis, and poorly defined nuclear–cytoplasmic boundaries. (B) Quantification of morphologically intact neurons in the striatum at ×50 magnification. Data are presented as the mean ± SD; *n* = 3 biological replicates per group with triplicate technical replicates. Quantification was performed on three randomly selected non‐overlapping striatal fields per section.

Representative striatal morphology is shown in Figure 3A. Neurons in the saline + saline group were densely and regularly arranged, with rounded cell bodies and clearly visible nuclei. In contrast, the APO + saline group showed wider intercellular spaces and a greater number of darkly stained, shrunken cells with pyknotic nuclei, consistent with neuronal injury. Quantification showed fewer morphologically intact neurons in the APO + saline group than in the saline + saline group (*p* < 0.001; Figure 3B). Treatment with CDRM, CDRH, or HAL increased the number of morphologically intact neurons relative to the APO + saline group (*p* = 0.009 or *p* < 0.001), suggesting attenuation of APO‐associated striatal injury.

### 3.4 Effects of CDR on Striatal Neurotransmitter Levels

**FIGURE 4:**
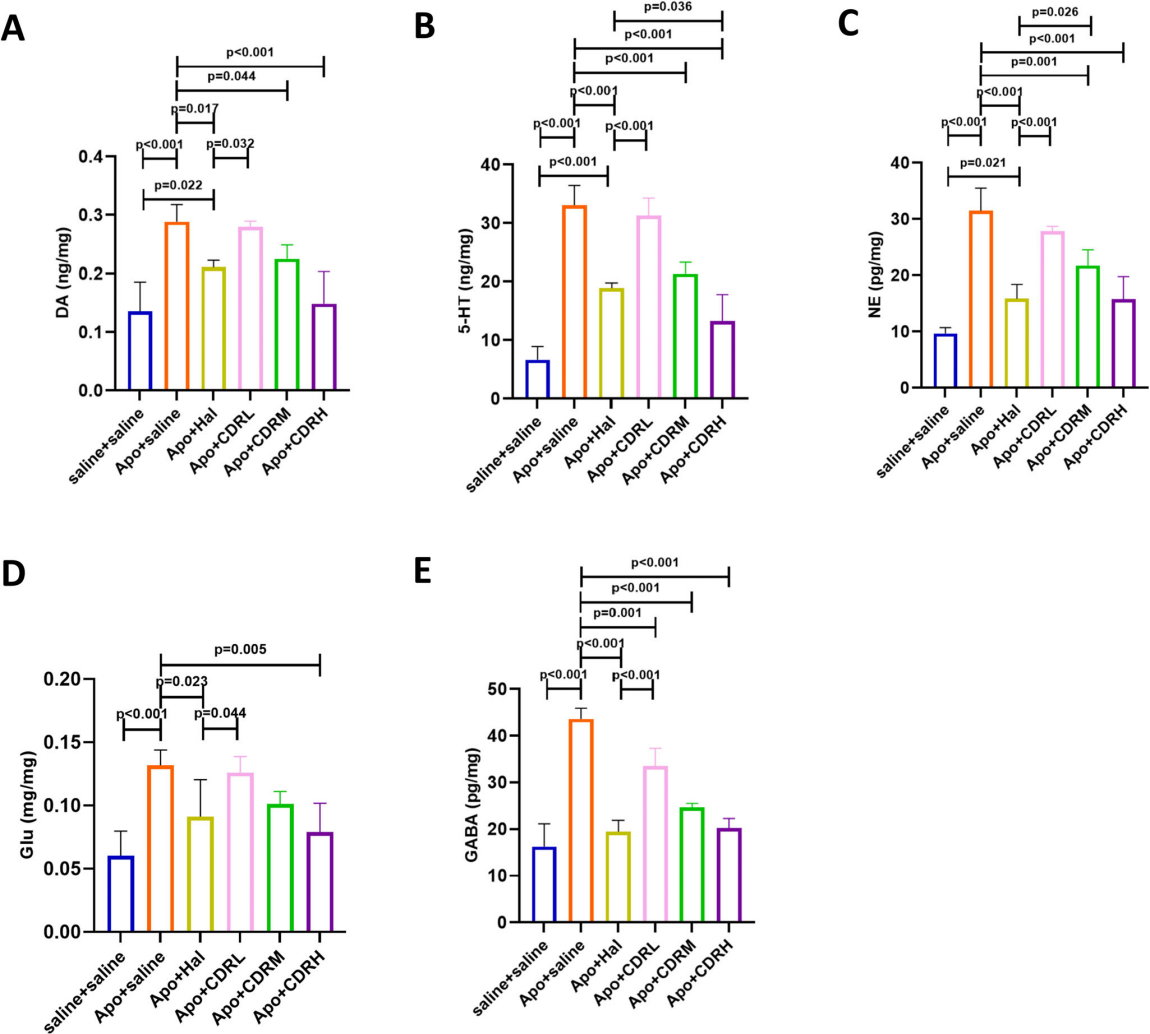
Striatal neurotransmitter concentrations in the experimental groups. (A) Dopamine (DA). (B) Serotonin (5‐HT). (C) Norepinephrine (NE). (D) Glutamate (Glu). (E) γ‐Aminobutyric acid (GABA). Data are expressed as mean ± SD. *n* = 3 biological replicates per group with triplicate technical replicates.

Striatal concentrations of DA, 5‐HT, NE, Glu, and GABA were significantly higher in the APO + saline group than in the saline + saline group (Figures 4A‐E; all *p* < 0.001). Compared with the APO + saline group, the APO + HAL and APO + CDRH groups showed significantly lower concentrations of all five neurotransmitters (Figures 4A‐E; *p* < 0.05 or *p* < 0.001). The APO + CDRM group also showed lower DA, 5‐HT, NE, and GABA concentrations (Figures 4A–C and 4E; *p* < 0.05 or *p* < 0.01). The 5‐HT concentration was lower in the APO + CDRH group than in the APO + HAL group (*p* = 0.036). Overall, the changes were more pronounced at higher CDR doses (Figures 4A‐E).

### 3.5 Network Pharmacology Analysis of CDR

**FIGURE 5:**
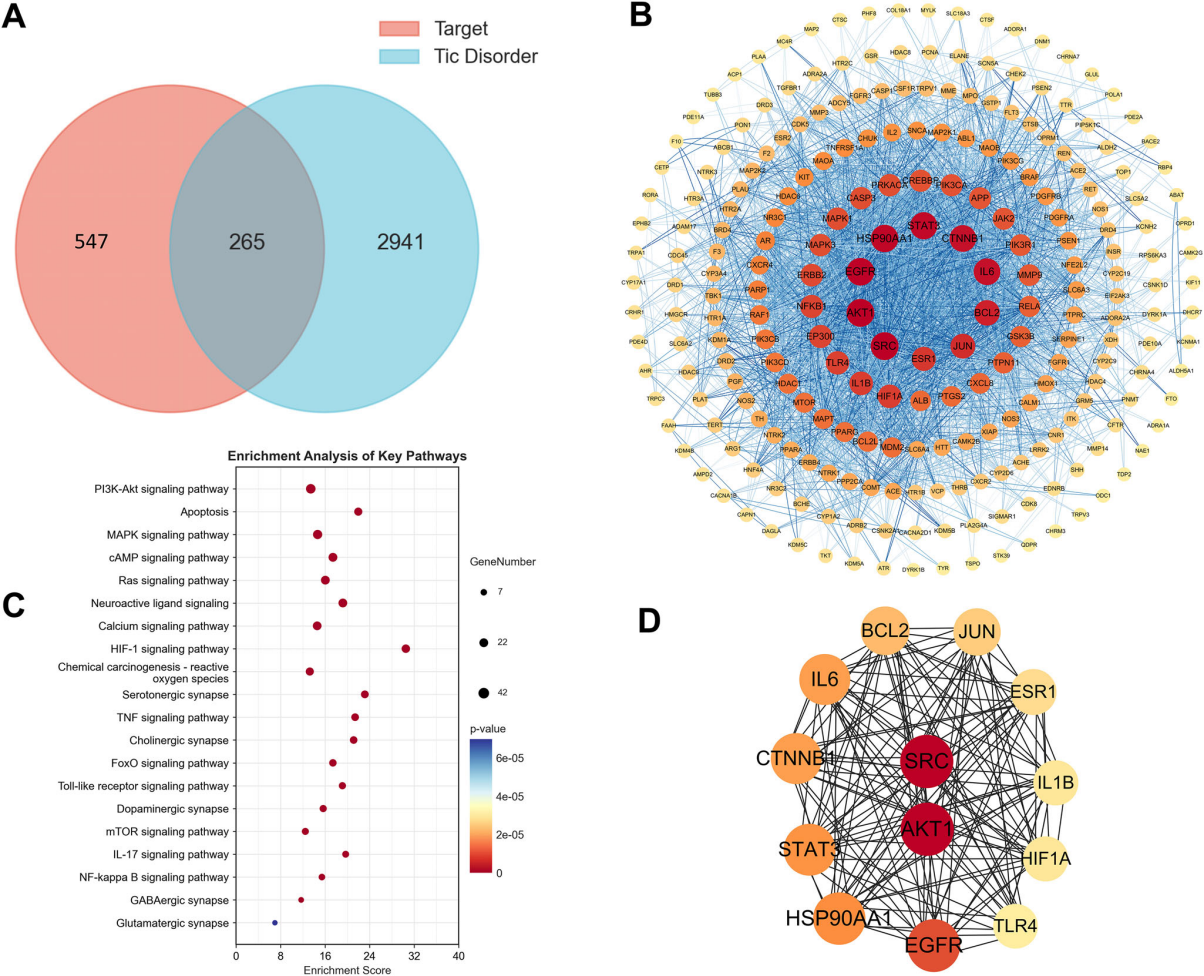
Network pharmacology analysis of XuanShi Choudong Recipe (CDR). (A) Venn diagram showing overlapping targets between CDR and tic disorder (TD). (B) Protein–protein interaction network of the common targets. (C) Bubble plot of significantly enriched pathways. (D) Highly ranked hub targets in the protein–protein interaction network. The analysis was performed in silico using public databases.

Among the 126 compounds identified by LC–MS, 71 met the prespecified pharmacokinetic criteria for predicted BBB permeability, high gastrointestinal absorption, and a bioavailability score ≥0.55. Putative targets were assigned to the retained compounds, and a compound–target interaction network was constructed (Figure S1). The network contained 336 nodes and 1236 edges. Licarin A, asarone, 6‐hydroxy‐2‐(4‐methoxyphenyl)‐4H‐chromen‐4‐one, and isocurcumenol had relatively high degree values, suggesting that they may contribute to the pharmacological activity of CDR. GeneCards and OMIM searches yielded 3206 nonredundant TD‐associated genes (Table S2). Intersection analysis identified 265 common targets between CDR‐associated and TD‐associated target sets (Figure 5A; Table S3). The protein–protein interaction network contained 239 nodes and 3060 edges (Figure 5B). Highly ranked hub genes included AKT serine/threonine kinase 1 (AKT1), SRC proto‐oncogene, non‐receptor tyrosine kinase (SRC), epidermal growth factor receptor (EGFR), heat shock protein 90 alpha family class A member 1 (HSP90AA1), signal transducer and activator of transcription 3 (STAT3), catenin beta 1 (CTNNB1), interleukin 6 (IL6), B‐cell lymphoma 2 (BCL2), Jun proto‐oncogene, AP‐1 transcription factor subunit (JUN), estrogen receptor 1 (ESR1), interleukin 1 beta (IL1B), hypoxia‐inducible factor 1 alpha subunit (HIF1A), and Toll‐like receptor 4 (TLR4) (Figure 5D). KEGG enrichment analysis mainly identified the PI3K–Akt signaling pathway, apoptosis, the MAPK, cAMP, Ras, calcium, and HIF‐1 signaling pathways among the enriched terms (Figure 5C).

### 3.6 Transcriptomic Changes Associated With CDR Treatment

**FIGURE 6:**
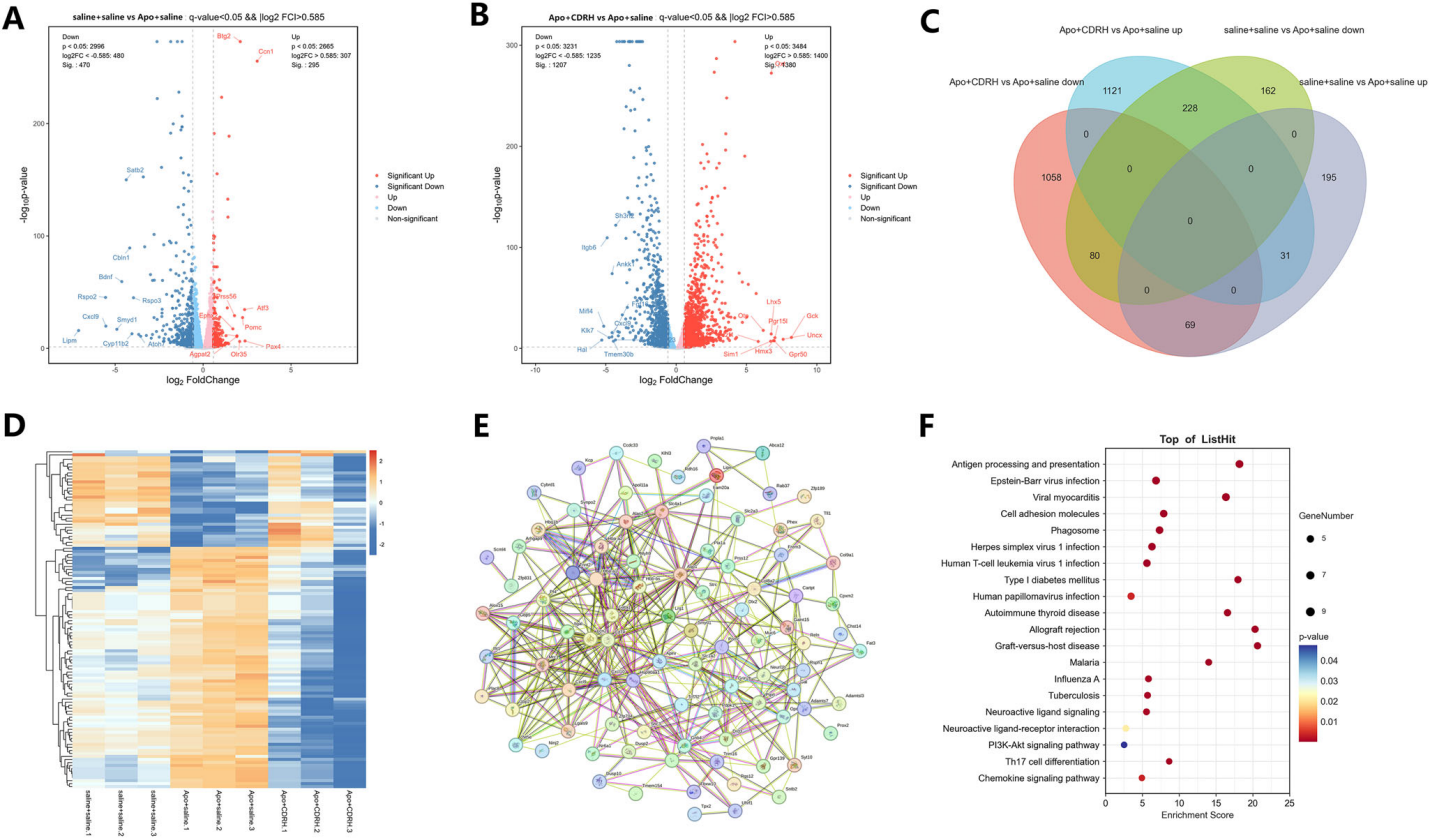
Transcriptomic analysis of genes and pathways associated with the effects of high‐dose CDR (CDRH) in the TD model. (A) Volcano plot of differentially expressed genes (DEGs) between the saline + saline and APO + saline groups. (B) Volcano plot of DEGs between the APO + CDRH and APO + saline groups. (C) Venn diagram identifying genes dysregulated by APO and oppositely regulated by CDRH. (D) Heatmap of the 111 reversibly regulated genes. (E) Protein–protein interaction network of the 111 genes. (F) KEGG pathway enrichment analysis of the 111 genes. Transcriptomic sequencing was performed using *n* = 3 biological replicates per group.

A total of 765 differentially expressed genes (DEGs) were identified between the saline + saline and APO + saline groups, including 295 upregulated and 470 downregulated genes (Figure 6A; Table S4). In addition, 2587 DEGs were identified between the APO + CDRH and APO + saline groups, including 1380 upregulated and 1207 downregulated genes (Figure 6B; Table S5). Among the genes dysregulated in the model group, 111 were regulated in the opposite direction after CDRH treatment, indicating partial reversal toward the expression pattern of the saline + saline group (Figure 6C). These genes were visualized in a heatmap (Figure 6D). A STRING‐based protein–protein interaction network of the 111 genes contained 95 nodes and 375 edges (Figure 6E). KEGG analysis showed enrichment in immune‐ and infection‐related pathways, including antigen processing and presentation, Epstein–Barr virus infection, and viral myocarditis, as well as the PI3K–Akt signaling pathway, chemokine signaling pathway, and T‐helper 17 cell differentiation (Figure 6F).

### 3.7 CDR Modulates the PI3K/AKT/Bcl‐2 Signaling Axis

**FIGURE 7:**
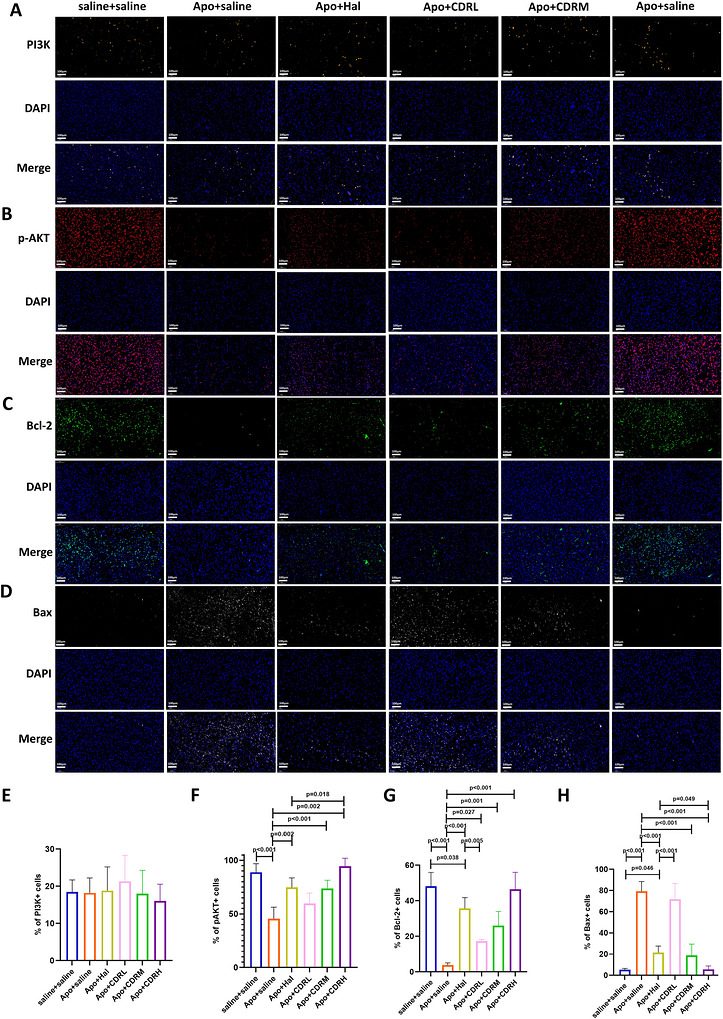
Immunofluorescence analysis of PI3K, p‐AKT, Bcl‐2, and Bax in the rat striatum (original magnification, ×20; scale bar = 100 µm). (A–D) Representative images of PI3K, p‐AKT, Bcl‐2, and Bax immunoreactivity, respectively. Fluorescence signals are pseudocolored for visualization, and nuclei are counterstained with DAPI. (E–H) Quantification of PI3K‐, p‐AKT‐, Bcl‐2‐, and Bax‐positive cells, respectively. Data are presented as the mean ± SD; *n* = 3 biological replicates per group, with triplicate technical replicates. Quantification was performed on three randomly selected non‐overlapping striatal fields per section.

On the basis of the network pharmacology and transcriptomic enrichment results, proteins in the PI3K/AKT/Bcl‐2 signaling axis were examined. Immunofluorescence analysis showed no significant between‐group difference in total PI3K immunoreactivity (Figures 7A **and** 7E). The percentage of p‐AKT‐positive cells was lower in the APO + saline group than in the saline + saline group (p < 0.001). This percentage increased after HAL or CDR treatment, with the largest increase observed in the CDRH group (Figures 7B **and** 7F; p = 0.002). The APO + saline group also showed a lower percentage of Bcl‐2‐positive cells and a higher percentage of Bax‐positive cells than the saline + saline group (Figures 7C and 7D; *p* < 0.001). HAL, CDRM, and CDRH treatment increased Bcl‐2 immunoreactivity and decreased Bax immunoreactivity. Bax immunoreactivity was lower in the CDRH group than in the APO + HAL group (*p* = 0.049).

**FIGURE 8:**
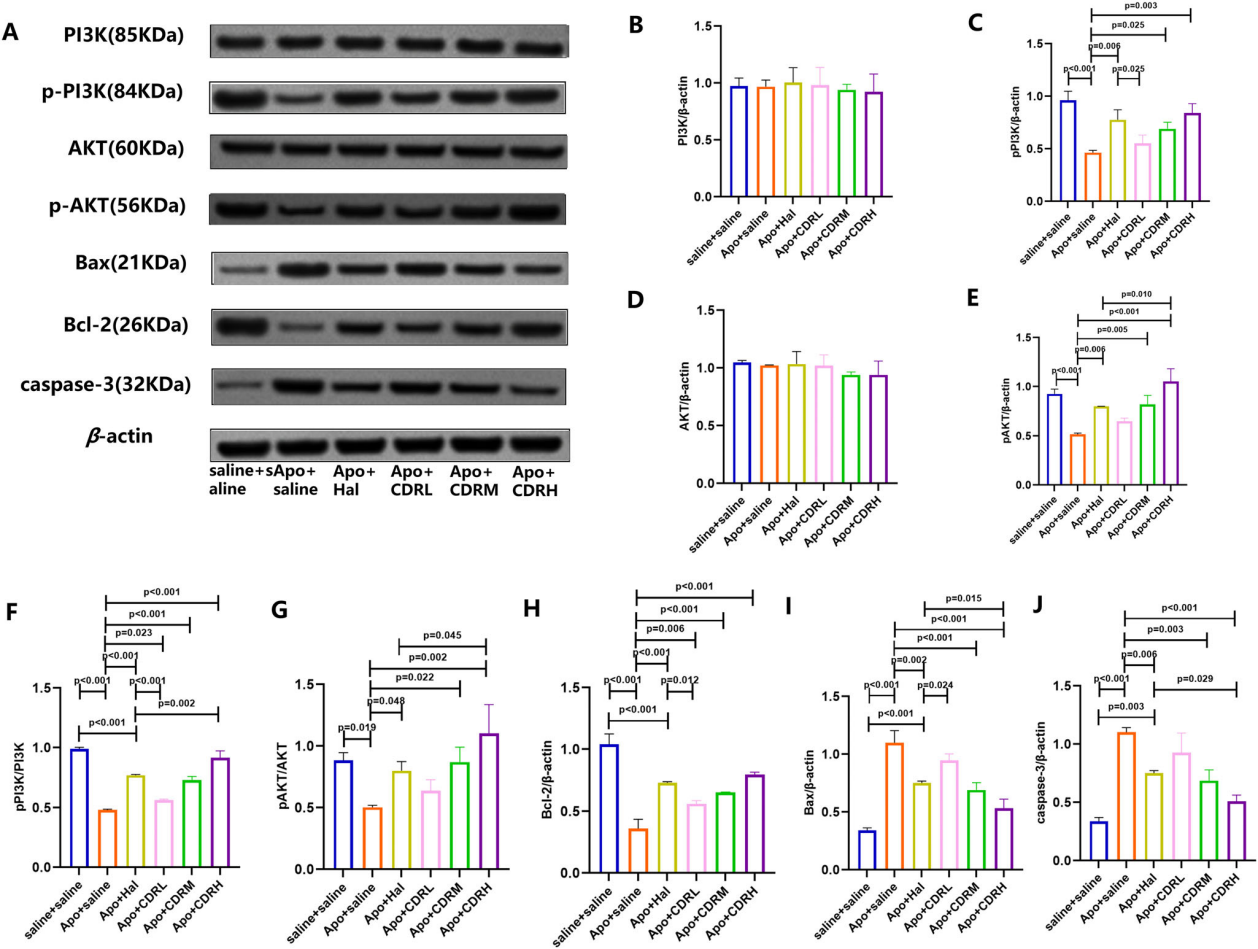
Western blot analysis of proteins in the PI3K/AKT/Bcl‐2 signaling axis in the rat striatum. (A) Representative bands for PI3K, p‐PI3K, AKT, p‐AKT, Bcl‐2, Bax, caspase‐3, and β‐actin. (B–J) Relative expression of PI3K, p‐PI3K, AKT, p‐AKT, the p‐PI3K/PI3K ratio, the p‐AKT/AKT ratio, Bcl‐2, Bax, and caspase‐3, respectively. Data are presented as the mean ± SD; n = 3 biological replicates per group, with triplicate technical replicates.

Western blotting provided concordant evidence. Total PI3K and AKT protein levels did not differ significantly among groups (Figures 8B **and** 8D). In contrast, the p‐PI3K/PI3K and p‐AKT/AKT ratios were lower in the APO + saline group and were increased in the HAL and CDR treatment groups (Figures 8F **and** 8G; *p* < 0.05 or *p* < 0.01), with values in the CDRH group approaching those in the saline + saline group. Bcl‐2 expression was reduced in the APO + saline group and increased after HAL or CDR treatment (Figure 8H; *p* < 0.05, *p* < 0.01, or *p* < 0.001). Conversely, Bax and caspase‐3 expression were elevated in the APO + saline group and decreased after CDR treatment, with more pronounced changes at the medium and high doses (Figures 8I **and** 8J; p < 0.01 or *p* < 0.001). Bax and caspase‐3 expression were lower in the CDRH group than in the APO + HAL group (*p* = 0.015 and *p* = 0.029, respectively). These findings indicate that CDR treatment was associated with enhanced PI3K/AKT phosphorylation and a shift toward an anti‐apoptotic protein‐expression profile.

### 3.8 CDR Attenuates Striatal Neuronal Apoptosis

**FIGURE 9:**
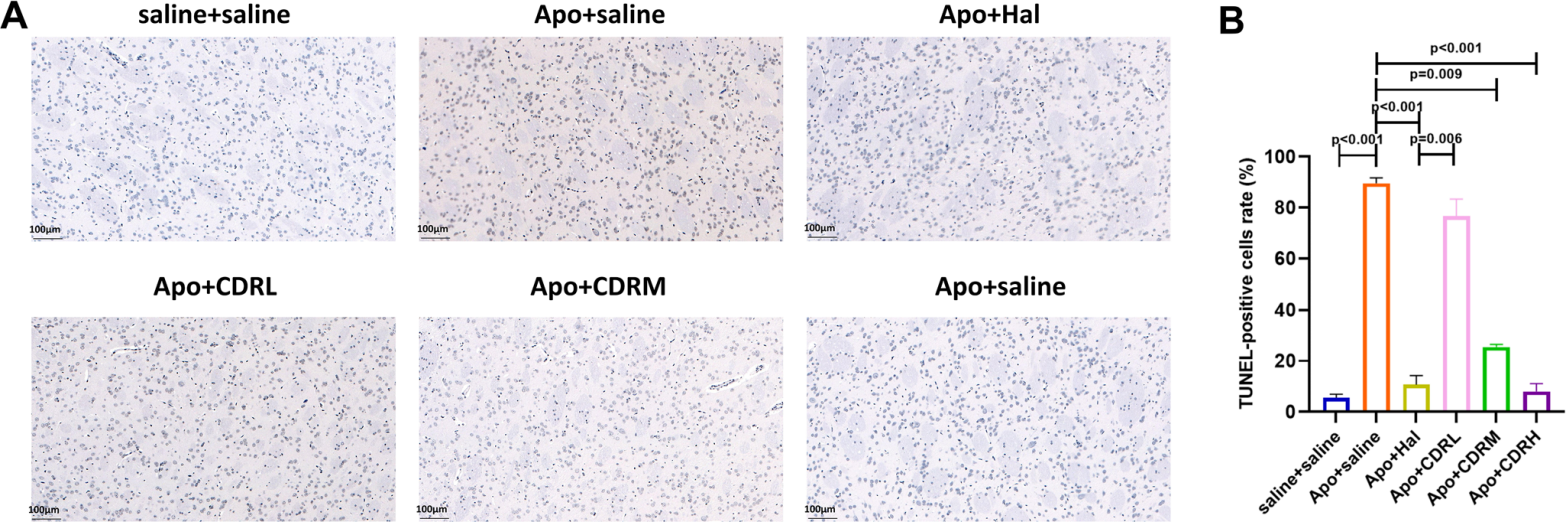
TUNEL staining of apoptotic cells in the rat striatum. (A) Representative images after CDR treatment (original magnification, ×20; scale bar = 100 µm). (B) Quantification of the percentage of TUNEL‐positive cells. Data are presented as the mean ± SD; *n* = 3 biological replicates per group, with triplicate technical replicates. Quantification was performed on three randomly selected non‐overlapping striatal fields per section.

TUNEL staining showed a higher percentage of TUNEL‐positive cells in the striatum of the APO + saline group than in the saline + saline group (*p* < 0.01; Figures 9A and 9B), indicating increased apoptosis‐associated DNA fragmentation. Compared with the APO + saline group, the percentage of TUNEL‐positive cells was lower after HAL, CDRM, or CDRH treatment (*p* < 0.01 or *p* < 0.001; Figure 9B).

### 3.9 CDR Alleviates Oxidative Stress in the Striatum

**FIGURE 10:**
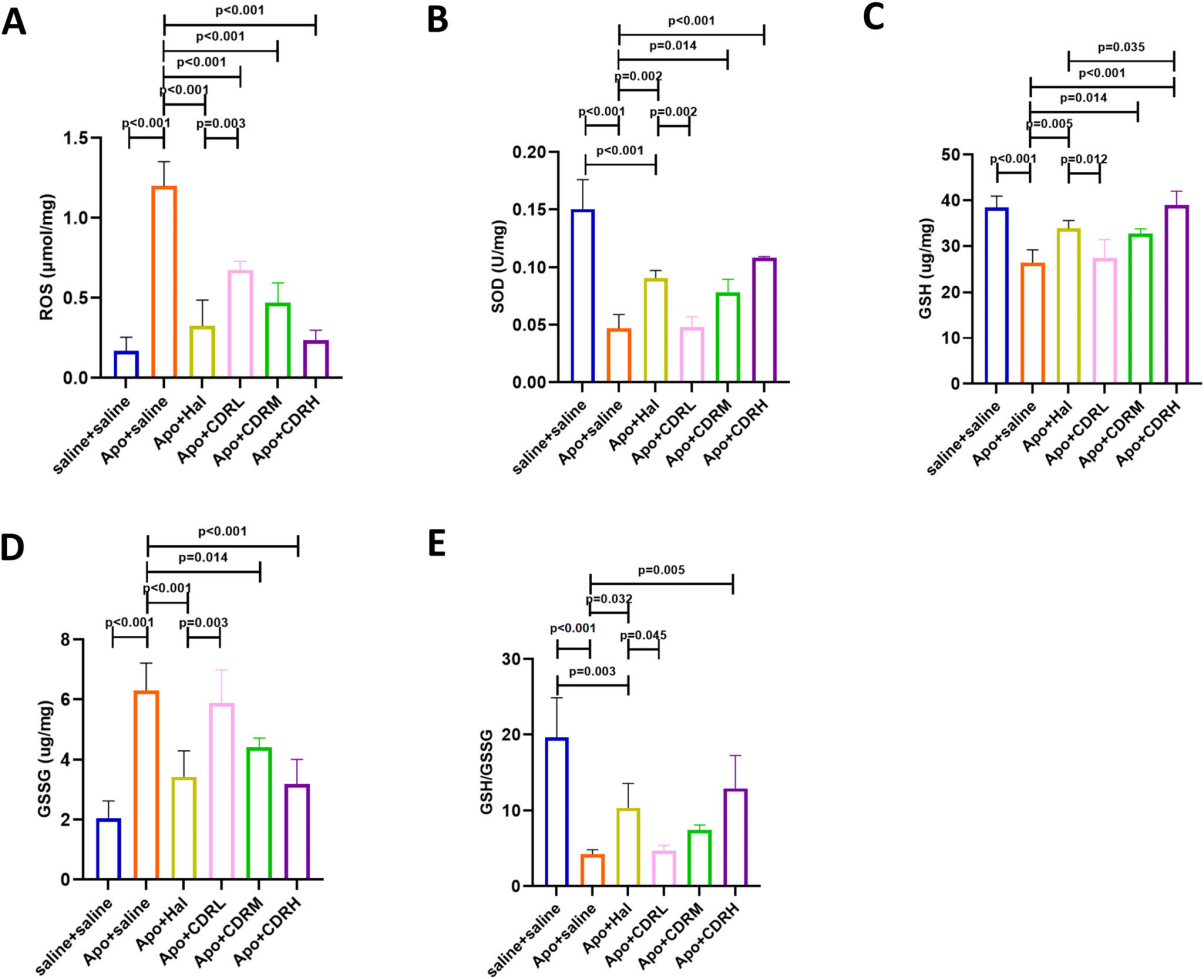
Oxidative stress markers in the rat striatum. (A) Reactive oxygen species (ROS). (B) Superoxide dismutase (SOD) activity. (C) Reduced glutathione (GSH). (D) Oxidized glutathione (GSSG). (E) GSH/GSSG ratio. Data are presented as the mean ± SD; *n* = 3 biological replicates per group, with triplicate technical replicates.

Striatal oxidative stress markers are shown in Figures 10A‐E. Compared with the saline + saline group, the APO + saline group had lower SOD activity (Figure 10B), lower GSH levels (Figure 10C), and a lower GSH/GSSG ratio (Figure 10E), together with higher ROS (Figure 10A) and GSSG (Figure 10D) levels (all *p* < 0.001). Relative to the APO + saline group, the treatment groups—particularly the APO + CDRH and APO + HAL groups—showed higher SOD activity, GSH levels, and GSH/GSSG ratios and lower ROS and GSSG levels (Figures 10A‐E) *p* < 0.05, *p* < 0.01, or *p* < 0.001).

## 4 Discussion

By integrating network pharmacology, transcriptomics, and in vivo experiments, this study investigated the preliminary pharmacological mechanisms associated with the effects of CDR in an APO‐induced rat model of tic‐like stereotypy. CDR reduced stereotypic behavior and hyperlocomotion and was associated with changes in striatal neurotransmitter levels, attenuation of oxidative stress and apoptosis, and increased PI3K and AKT phosphorylation. These findings support, but do not establish, the involvement of the PI3K/AKT/Bcl‐2 signaling axis in the observed neuroprotective effects. Dopamine receptor antagonists are commonly used to manage clinically significant tics. Haloperidol is effective in reducing tic severity, but its clinical use—particularly in children—may be limited by adverse effects such as sedation, extrapyramidal symptoms, and tardive dyskinesia (Cothros et al. 2019; Roessner et al. 2022). Because TD is a heterogeneous neurodevelopmental disorder involving multiple neurotransmitter systems and neural circuits, interventions directed at a single target may not adequately address all clinical manifestations (Ganos 2016). TCM formulas are therefore of interest as multicomponent interventions that may act through several biological processes, although their active constituents and causal mechanisms require rigorous validation (Lu et al. 2025; Zhang et al. 2025). CDR is an empirical formula derived from Xuan's pediatrics tradition. Preliminary clinical observations have reported reductions in tic severity and acceptable tolerability in preschool children, and earlier animal work suggested effects on neurotransmitter concentrations and dopamine receptor expression (Xu et al. 2025; Xuan et al. 2017; Yang et al. 2018). The present study extends these observations by combining chemical profiling, systems‐level prediction, transcriptomic analysis, and targeted experimental validation.

In the open‐field test and stereotypic behavior assessment, medium‐ and high‐dose CDR reduced APO‐associated hyperlocomotion and stereotypy. HAL produced similar reductions in locomotor variables, in some cases to values below those of the control group, which is consistent with its known sedative effects (Roessner et al. 2022). Reduced locomotion can reflect improvement in APO‐induced hyperactivity, nonspecific sedation, or both. Accordingly, the behavioral findings should be interpreted as evidence that CDR attenuated the measured tic‐like phenotypes rather than as definitive proof of a specific anti‐tic mechanism. LC–MS profiling yielded 126 putatively identified compounds, predominantly phenylpropanoids and polyketides, benzenoids, and lipid‐related molecules. Network pharmacology highlighted several candidate targets, including AKT1 and BCL2, and identified enrichment of the PI3K–Akt signaling pathway. Transcriptomic analysis further showed that CDRH oppositely regulated 111 genes that were dysregulated in the model group, with enrichment of the PI3K–Akt pathway among the associated terms. These convergent findings motivated targeted analysis of PI3K/AKT/Bcl‐2‐related proteins. CDR increased the p‐PI3K/PI3K and p‐AKT/AKT ratios, increased Bcl‐2 expression, and decreased Bax and caspase‐3 expression. These molecular changes occurred alongside fewer TUNEL‐positive cells and improved neuronal morphology, suggesting an association between CDR treatment and reduced apoptosis. PI3K/AKT signaling is an important regulator of cell survival and oxidative‐stress responses in several tissue‐injury models (Qin et al. 2021; Wang et al. 2020). Genetic and epigenetic studies also implicate PI3K–AKT‐related processes in TD. Whole‐exome sequencing in a Chinese Han cohort identified the PI3K–AKT pathway as a candidate risk pathway (Lu et al. 2024), whereas an epigenome‐wide association study in Korean children reported differential methylation in genes linked to PI3K–AKT and MAPK signaling (Ko et al. 2024). These studies are broadly consistent with the pathway enrichment observed here, but they do not demonstrate that PI3K/AKT signaling mediates the therapeutic effect of CDR. Network pharmacology and transcriptomics further suggested that multiple CDR constituents and targets may be involved. Functional studies are required to determine whether these targets act independently or cooperatively. KEGG enrichment analysis predominantly identified several signaling pathways, including the PI3K‐Akt pathway, apoptosis, T17, MAPK, cAMP, Ras, calcium signaling, and HIF‐1 signaling pathways. The core targets involve AKT1, HIF1A, BCL2, IL6, IL1B, TLR4, HSP90AA1, STAT3, and JUN. These targets and pathways are intricately linked to the regulation of oxidative stress and encompass fundamental pathological processes of TD, such as neuroinflammation and dysregulated dopamine signaling. Neuroimmune molecules and cells, including TH17 and TNF‐α, are closely linked to the disruption of the blood‐brain barrier, the activation of striatal microglia, and the initiation of neuroinflammatory cascades (Sun and Bai 2026). Animal studies have further demonstrated that the expression levels of TLR4, IL‐6, interleukin 8 (IL‐8), and tumor necrosis factor‐α (TNF‐α) in the striatum of rats in the TD group were significantly elevated compared to those in the striatum of rats in the control group (Liu et al. 2026). HIF1A, JUN, and HSP90AA1 are pivotal components of the oxidative stress regulatory network. Prior research has demonstrated that, under TD conditions, an imbalance in the oxidative‐antioxidant system—marked by the accumulation of ROS and a reduction in endogenous antioxidant capacity—contributes to neuronal damage in striatal neurons (Fichna et al. 2023; Long et al. 2019). The intracellular signaling pathways of cAMP, Ras, and calcium, which operate downstream of dopamine receptors, are essential; their dysfunction not only leads to abnormal striatal synaptic plasticity and imbalances in neuronal excitability but also exacerbates oxidative stress through calcium overload and mitochondrial dysfunction (Albin and Mink 2006; Nakano et al. 2010; Singer and Minzer 2003). Collectively, these multi‐target, multi‐pathway regulatory networks form the biological foundation for the efficacy of CDR in the treatment of tic disorders. These signaling pathways are not exclusive to TD; rather, they play a broad role in neurodevelopment, inflammation, cell survival, and synaptic plasticity. Additionally, their functions may differ across various developmental stages. Neuroimaging studies reveal age‐related differences in striatal and cortico‐striato‐thalamo‐cortical circuitry associated with TD (Hsu et al. 2020; Jin et al. 2026). Because juvenile rats were used in the present study, the observed molecular changes may be more relevant to childhood‐onset TD than to persistent adult disease. Direct developmental comparisons will be needed to test this possibility.

Altered central neurotransmission, particularly involving DA, 5‐HT, GABA, Glu, and NE, has been implicated in TD (Qian et al. 2022). In the present model, striatal concentrations of all five neurotransmitters were elevated, and CDR shifted these concentrations toward control values in a dose‐related manner. Dopaminergic hyperactivity and altered dopamine receptor sensitivity are among the most established neurochemical hypotheses of TD, although the direction and regional specificity of dopamine abnormalities may vary (Wang et al. 2023). The reduction in striatal DA after CDR treatment may therefore contribute to the improvement in APO‐induced stereotypy, but the present data do not identify the upstream molecular target responsible for this change. HAL also reduced striatal DA, consistent with its antagonism of dopamine receptors and consequent changes in dopamine turnover (Greydanus and Tullio 2020; Mogwitz et al. 2018). The elevated GABA concentration in the APO + saline group differs from a simple GABA‐deficiency model of TD (Singer and Augustine 2019). One possible explanation is compensatory enhancement of GABAergic tone in response to chronic dopaminergic stimulation and excessive excitatory drive. Dopamine–GABA interactions in the striatum are complex and can induce adaptive synaptic and extrasynaptic changes (Ali et al. 2023; Tritsch and Sabatini 2012). Clinical magnetic resonance spectroscopy studies have likewise reported regionally increased GABA in some patients with Tourette syndrome, potentially reflecting compensatory inhibition of motor excitability (Draper et al. 2014). Thus, the decrease in GABA after CDR treatment should not necessarily be interpreted as direct suppression of GABAergic transmission; it may instead reflect partial normalization of network activity as dopaminergic and glutamatergic drive decreases. The changes in NE, 5‐HT, and Glu further support the involvement of multiple neurotransmitter systems. Elevated cerebrospinal‐fluid NE has been reported in Tourette syndrome, and experimental manipulation of brain NE can alter locomotor activity (Jones and Hess 2003) (Leckman et al. 1995). Increased serotonergic activity has also been described in selected brain regions and clinical subgroups, although findings are not uniform. Excessive Glu release may increase excitatory drive within cortico‐striato‐thalamo‐cortical circuits and contribute to abnormal motor output (Nordstrom et al. 2015). The concurrent behavioral improvement and broad neurotransmitter changes observed after CDR treatment are compatible with partial restoration of striatal excitation–inhibition balance; however, region‐specific neurochemical measurements and receptor‐level experiments are needed to test this interpretation.

Several limitations should be considered. First, the APO‐induced rat model is useful for preliminary assessment of dopamine‐related stereotypy but does not reproduce the full clinical spectrum of TD, including vocal tics, comorbid neuropsychiatric symptoms, chronic developmental trajectories, or the complete dysfunction of cortico‐striato‐thalamo‐cortical circuitry (Ganos 2016; Nordstrom et al. 2015). Extrapolation to pediatric patients should therefore be made cautiously. Second, the proposed involvement of the PI3K/AKT/Bcl‐2 signaling axis is based on convergent but correlative evidence. Although pathway‐related protein changes were observed alongside neuroprotective phenotypes, causality was not tested using pathway inhibitors, activators, or targeted genetic manipulation. Such gain‐ and loss‐of‐function studies are required before this pathway can be considered a mediator of CDR activity. Third, network pharmacology is an in silico predictive approach. Predicted gastrointestinal absorption, BBB permeability, and bioavailability may not reflect in vivo exposure, biotransformation, or brain penetration after oral administration of a complex decoction. In addition, LC–MS/MS annotation relied mainly on spectral‐library matching, so some constituents remain putatively identified—their exact botanical or chemical source, potentially obscured by decoction‐generated, processing, or matrix‐interference signals, could not be fully determined; future work should validate representative compounds with authentic reference standards, characterize their plasma and brain exposure after CDR administration, and conduct targeted analyses to clarify the identity and source of such constituents. We also acknowledge that the network pharmacology analysis presented here remains primarily descriptive and hypothesis‐generating—experimental validation of the predicted targets, using pharmacological or genetic approaches, will be required in future studies to confirm their functional relevance. Fourth, although the bioinformatic analyses suggested the involvement of multiple targets and pathways, their functional interactions and potential synergy were not experimentally tested. The animal sample size was also limited, particularly for exploratory molecular and biochemical outcomes (*n* = 3), which reduces statistical precision and generalizability. Multi‐platform analyses may additionally introduce batch‐related variability. Future studies should use etiologically complementary TD models, larger independently replicated cohorts, prespecified causal experiments, and pharmacokinetic analyses of brain‐penetrant constituents. Clinical studies will ultimately be required to determine the relevance of these findings to patients.

In conclusion, CDR attenuated APO‐induced tic‐like behaviors in rats and was associated with shifts in striatal neurotransmitter concentrations, reduced oxidative stress and apoptosis, and increased phosphorylation of PI3K and AKT together with changes in Bcl‐2, Bax, and caspase‐3 expression. These findings provide preliminary experimental support for the hypothesis that the PI3K/AKT/Bcl‐2 signaling axis may contribute to the effects of CDR. Further mechanistic and translational studies are required to establish causality, identify the active brain‐penetrant constituents, and determine clinical relevance.

## Author Contributions

J. H., J. M., Y. C., and Y. P. conceived and designed the study, acquired and analyzed the data, and drafted the manuscript. Y. C., J. X., and D. C. performed the experiments and contributed to data interpretation. J. C. and X. X. supervised the study, critically revised the manuscript, and approved the final version. All authors read and approved the final manuscript.

## Funding

This work was supported by the Hangzhou Xuan's Pediatrics Academic Inheritance Studio Construction Project (no. [2012]228), the Chen Jian Famous Senior Chinese Medicine Expert Inheritance Studio (GZS2021023), and the Zhejiang Provincial TCM Science and Technology Program Special Project for TCM Modernization (2021ZX006).

## Ethics Statement

The study protocol was approved by the Institutional Animal Care and Use Committee of Zhejiang Chinese Medical University (approval no. IACUC‐20220822‐07). All animal procedures were conducted in accordance with the institutional guidelines for the care and use of laboratory animals. The study did not involve human participants; therefore, informed consent was not applicable.

## Conflicts of Interest

The authors declare no conflicts of interest.

## Supporting information




**Supplementary Table**: brb371687‐sup‐0001‐SuppMat.xls

**Supplementary Material**: brb371687‐sup‐0002‐SuppMat.docx

## Data Availability

The data generated or analyzed during this study are included in the published article and its supplementary materials. Additional data are available from the corresponding authors upon reasonable request.
