# Supplementary material for: Integrative Network Pharmacology and Transcriptomics Reveal the Mechanism of XuanShi Choudong Recipe in the Treatment of Tic Disorder
Source: Brain Behav. 2026 Aug 17;16(8):e71687. doi: 10.1002/brb3.71687 (PMC13482021; doi:10.1002/brb3.71687)
Supplement: Supplementary file 1 [file BRB3-16-e71687-s002.docx]

**Supplementary Table of Contents**

This supplementary file contains the following materials, each cross-referenced to the corresponding section of the main text for easy navigation.

Supplementary Methods

Supplementary Methods 1 — Detailed procedures for transcriptome sequencing and bioinformatic analysis (corresponds to Section 2.6)

S1.1 RNA extraction and quality control

S1.2 Library construction and high-throughput sequencing

S1.3 Raw data quality control and genome alignment

S1.4 Transcript assembly and differential expression analysis

S1.5 Functional enrichment analysis

Supplementary Tables

Table S1 — Main chemical components identified in the aqueous extract of CDR (corresponds to Section 3.1)

Table S2 — TD-associated target genes (corresponds to Sections 3.5)

Table S3 — Overlapping targets between CDR and TD (corresponds to Section 3.5)

Table S4 — DEGs between the model and control groups (corresponds to Section 3.6)

Table S5 — DEGs between the CDRH and model groups (corresponds to Section 3.6)

Supplementary Figures

Figure S1: Herb-compound-target network (corresponds to Section 3.5)

**Supplementary Methods 1**. Detailed procedures for transcriptome sequencing and bioinformatic analysis

### S1.1 RNA extraction and quality control

Striatal tissue samples were rapidly ground into fine powder in liquid nitrogen, and total RNA was extracted using TRIzol reagent following the manufacturer’s standard protocol. RNA concentration and purity were determined using a NanoDrop 2000 spectrophotometer (Thermo Scientific, USA), and RNA integrity was assessed using an Agilent 2100 Bioanalyzer (Agilent Technologies, USA). Only samples meeting library construction quality criteria were included. Three biological replicates were used per group, with a total of 12 samples included in the sequencing analysis.

### S1.2 Library construction and high-throughput sequencing

mRNA sequencing libraries were constructed using the VAHTS Universal V6 RNA-seq Library Prep Kit according to the manufacturer’s instructions. Briefly, poly(A)-tailed mRNA was enriched using Oligo(dT)-coated magnetic beads, followed by fragmentation, first-strand cDNA synthesis using random hexamer primers and M-MuLV reverse transcriptase, and second-strand synthesis using RNase H and DNA polymerase I. The resulting double-stranded cDNA underwent end repair, A-tailing, and adapter ligation. Fragments of approximately 300 bp were selected using AMPure XP beads and amplified by PCR with sample-specific index primers. Library quality was verified using an Agilent 2100 Bioanalyzer, and library concentration was quantified by qPCR with a threshold of >2 nM. Qualified libraries were pooled in equimolar amounts and sequenced on an Illumina/BGI platform in paired-end mode.

### S1.3 Raw data quality control and genome alignment

Raw sequencing reads were processed using fastp to obtain high-quality clean reads. Filtering criteria included removal of adapter-contaminated reads, removal of reads with ambiguous bases (N content > 0.2%), and removal of reads in which low-quality bases (Q ≤ 20) accounted for more than 50% of total read length. Quality metrics including Q30 score, GC content, and clean data yield were calculated for each sample. A total of 96.61 Gb of clean data was obtained from 12 samples, with ≥4.20 Gb per sample and Q30 ≥ 96.01%, indicating high sequencing quality. Clean reads were aligned to the Rattus norvegicus reference genome (Rnor_6) using STAR. Mapping rates ranged from 97.14% to 97.73% across all samples. Saturation analysis confirmed sufficient sequencing depth for downstream analyses.

### S1.4 Transcript assembly and differential expression analysis

Transcript assembly and merging were performed using StringTie based on genome alignment results. Gene-level read counts were generated using HTSeq (union mode). Differential expression analysis was conducted using the DESeq2 R package, which models count data using a negative binomial distribution.

Gene expression normalization, dispersion estimation, and hypothesis testing were performed within DESeq2. The Wald test was used to evaluate differential expression between groups. To control for multiple testing, raw P values were adjusted using the Benjamini–Hochberg (BH) method to control the false discovery rate (FDR). Genes with an absolute log₂ fold change > 0.585 and FDR-adjusted P value < 0.05 were defined as significantly differentially expressed genes. FPKM values were calculated for visualization purposes only and were not used for statistical inference.

### S1.5 Functional enrichment analysis

Functional enrichment analysis of differentially expressed genes was performed using the clusterProfiler R package. Gene Ontology (GO), Kyoto Encyclopedia of Genes and Genomes (KEGG), and Reactome pathway analyses were conducted based on over-representation analysis (ORA). Statistical significance was assessed using a hypergeometric test. The background gene set was defined as all genes detected in the differential expression analysis that could be mapped to the corresponding annotation databases. To account for multiple hypothesis testing, enrichment P values were adjusted using the Benjamini–Hochberg procedure, and results were reported as FDR-adjusted P values.

GO terms (biological process, cellular component, molecular function), KEGG pathways, and Reactome pathways were analyzed separately, with FDR < 0.05 considered statistically significant enrichment. Disease Ontology (DO) analysis was performed using the same statistical framework where applicable. All bioinformatic analyses were performed on the OECloud platform (https://cloud.oebiotech.com).

**Figure S1.** The Herb-compound-target network.
